# Light-Enhanced Microbial Organic Carbon Yield

**DOI:** 10.3389/fmicb.2017.02157

**Published:** 2017-11-16

**Authors:** John R. Casey, Sara Ferrón, David M. Karl

**Affiliations:** Center for Microbial Oceanography, School of Ocean and Earth Science and Technology, University of Hawaii at Manoa, Honolulu, HI, United States

**Keywords:** photoheterotrophy, glycolate, substrate assimilation efficiency, photorespiration, diel cycles of microbial metabolism

## Abstract

Molecular evidence for proteorhodopsin- and bacteriochlorophyll-based photoheterotrophy is widespread in oligotrophic marine microbial community metagenomes, and has been implicated in light-enhanced growth rates, substrate uptake rates, and anapleurotic carbon fixation, thus complicating the web of interactions within the ‘microbial loop.’ We quantified photoheterotrophic metabolism of the oxidized organic acid glycolate, a fast-turnover and exclusively phytoplankton-derived substrate at an oligotrophic site in the subtropical North Pacific Ocean. As expected, concentration-dependent changes in uptake rates were observed over the diel cycle, with maxima occurring at midday. Although no light-enhanced substrate uptake rates were observed, samples exposed to light altered the balance between assimilation and respiration, resulting in an approximately four-fold increase in glycolate-specific assimilation efficiency. Energy demand for such a metabolic adjustment was linearly related to light, consistent with photoheterotrophy.

## Introduction

The web of microbially mediated transformations of carbon and energy in the oceans is intricate and dynamic. The conduit through which a great majority of oceanic respiration is channeled is the dissolved organic matter (DOM) reservoir. DOM is composed of thousands of unique molecules in widely varying concentrations (Mopper et al., 2007), and the spectrum of turnover spans from minutes (Fuhrman and Ferguson, 1986) to millennia (Ziolkowski and Druffel, 2010; Zigah et al., 2017). Among other physicochemical attributes, the thermodynamic properties of organic substrates governs their turnover, with high enthalpy substrates supporting sub-optimal microbial growth rates and therefore turning over more slowly than low enthalpy substrates (Casey et al., 2015). However, since the discovery of two unique light-harvesting systems widespread in marine bacteria and archaea, aerobic anoxygenic phototrophy (AAP; Shiba et al., 1979) and proteorhodopsin (PR) phototrophy (PRP; Béjà et al., 2000), the traditional view of a primary producer-DOM-secondary producer microbial loop (Azam et al., 1983) should be revised (Karl, 2014). Collectively, PR and aerobic anoxygenic phototrophic bacteria and archaea comprise most of the total heterotrophic microbial community in oligotrophic marine ecosystems (Rusch et al., 2007), and PRs have been found in diverse bacterial phyla (McCarren and DeLong, 2007), including the numerically dominant alphaproteobacterium SAR11 and marine archaea (Frigaard et al., 2006). While nutrient and ion transport have been associated with rhodopsins (Chan et al., 1981; Dimroth, 1990; Feng et al., 2013; Inoue et al., 2013; Kwon et al., 2013; Yoshizawa et al., 2014), both PR and the bacteriochlorophyll (BChl) complex are capable of generating a proton motive force(pmf) to supplement the ATP demands of biosynthetic and maintenance functions. The PR pmf generated has been shown in monoclonal cultures to markedly stimulate growth rates (Gómez-Consarnau et al., 2007; Kimura et al., 2011; Palovaara et al., 2014), anapleurotic carbon fixation rates (Palovaara et al., 2014), substrate uptake rates (Alonso-Saez et al., 2006; Michelou et al., 2007; Mary et al., 2008), and to resuscitate carbon-starved cells (Gómez-Consarnau et al., 2010; Steindler et al., 2011). Whether the high abundance and diversity of AAP and PRP in the marine environment indicates a physiological cost-benefit solution to energy limitation of heterotrophic microbial growth on low-yield, thermodynamically efficient substrates remains unclear.

The PRP and AAP pmf may provide microbes with a reliable energy source to supplement, or perhaps to partly relieve oxidative phosphorylation demands (Johnston et al., 2005). Indeed, Koblížek et al. (2010) measured a 70% reduction in respiration rates of an AAP *Roseobacter* isolate when grown on glutamate as a sole carbon source in the presence of light. Accordingly, the ‘shaft work’ provided to facultative photoorganoheterotrophs by photochemical energy transduction should decouple substrate chemical energy potential from the anabolic yields of obligate chemoorganoheterotrophs (von Stockar et al., 2006). We hypothesized that light repression of photoorganoheterotrophic respiration improves substrate-specific growth yields within natural microbial assemblages, especially for substrates more oxidized than their anabolic end-products.

Light-enhanced nucleic acid and amino acid assimilation rates have been reported in whole communities (Church et al., 2004, 2006) and within flow cytometry sorted populations of *Prochlorococcus, Synechococcus*, and small, low nucleic acid bacterioplankton, including SAR11 (Michelou et al., 2007; Mary et al., 2008; Gómez-Pereira et al., 2012). However, sorted populations have not been directly linked to their respective respiration rates, and thus evidence for light-enhanced bacterial growth efficiency has necessarily been inferred from bulk oxygen consumption (Cottrell et al., 2008). A suitable substrate to evaluate light-enhanced heterotrophic growth yield is the hydroxy acid glycolate. Glycolate has long been recognized to be an important light-dependent excretory product of phytoplankton (Tolbert and Zill, 1957; Nalewajko et al., 1963), and featured at the center of a lively debate regarding its extracellular production (Sharp, 1977; Mague et al., 1980; Fogg, 1983; Bjørnsen, 1988) and consumption by heterotrophic bacteria (Wright and Shah, 1977). Glycolate is secreted as a result of photorespiration from photoautotrophic microbes like high-light adapted *Prochlorococcus* strains (Bertilsson et al., 2005), some of which lack a complete salvage pathway (Casey et al., 2016). Since photorespiration is likely the sole extracellular source of glycolate, and since glycolate permease transporters and glycolate oxidases and dehydrogenases are present in SAR11, glycolate represents a direct transfer of oxidized, newly fixed photosynthate to support heterotrophic carbon and energy demands. In this study, radiorespirometry experiments were conducted to determine the concentration-dependent kinetics of glycolate uptake, the diel cycle of glycolate uptake, and the effect of light within that cycle and as a function of depth within the euphotic zone (5–100 m).

## Materials and Methods

### Station Locations and Sample Collection

Samples were collected on two separate expeditions (Cruise 1 – September 2013 at 22° 75′ N, 158° 00′ W and Cruise 2 – July–August 2015 at 24° 25′ N, 156° 45′ W) in the North Pacific Subtropical Gyre north of the island of O’ahu. The kinetics experiment was carried out during Cruise 1. Cruise 2 followed a semi-Lagrangian track near the center of an anticyclonic mode-water eddy feature, facilitated by maintaining ship’s position with World Ocean Circulation Experiment Surface Velocity Profile drifters with 15 m-depth drogues. Sampling for glycolate tracer incubation time-series was conducted at 4 h intervals, uninterrupted over the course of 2 days. Additionally, a depth profile of glycolate uptake rates was conducted using a surface-tethered array, designed to accommodate sample bottles suspended at 5, 25, 50, 75, and 100 m, which were deployed from dawn to dusk on the last day of the time-series experiments. Water samples were collected using polyvinyl chloride Niskin^®^ bottles mounted on a rosette equipped with dual conductivity, temperature, pressure, and oxygen sensors, a transmissometer, and a triplet fluorometer (SBE 911plus, Sea-Bird Electronics, Inc.). Photosynthetically active radiation (PAR; 400–700 nm wavelength band) are measured both at the surface in time-series experiments by shipboard quantum cosine sensor (LI-190R; LI-COR Inc.) with data logger (LI-1000; LI-COR Inc.), and also in depth profiles (0–190 m) by a free-falling profiling hyperspectral radiometer (HyperPro, Satlantic LP.). Incubation time-integrated PAR was calculated by scaling shipboard PAR to incubator transmittance (50%).

### Community Stocks, Production, and Respiration Data

Alongside glycolate incubations, samples were collected for chlorophyll a (Chl a), primary production (PP), and microbial community respiration (MCR). Chl a and PP measurements were conducted according to Hawaii Ocean Time-series standard protocols (Karl and Dore, 2001^1^). Briefly, for Chl a, 125 ml samples were pressure filtered onto 25 mm glass fiber filters (Whatman GF/F) and stored in acetone at -20°C until analyzed fluorometrically. For PP, 500 ml samples were collected in triplicate at different depths and incubated *in situ* on a surface-tethered array deployed before sunrise and recovered after sunset. Prior to deployment, bottles were spiked with H^14^CO_3_^-^ to yield a final radioactivity of approximately 2 MBq L^-1^. After a 14 h incubation, samples were filtered onto GF/F filters, acidified in glass scintillation vials with 1 ml 2M hydrochloric acid and allowed to vent for 24 h prior to the addition of 10 ml Ultima Gold LLN cocktail and liquid scintillation counting. Similarly, samples for gross oxygen production (GOP) and MCR were collected in triplicate in 125 ml Pyrex glass bottles, spiked with H_2_^18^O (Medical Isotopes, 97.2% ^18^O) to a final δ^18^O(H_2_O) of approximately 2300‰, and incubated *in situ* along with the PP array. After recovery, biological activity was stopped by adding 100 μL of saturated mercuric chloride solution. In addition, triplicate time-zero samples were collected at each depth and poisoned at the beginning of the deployment. Mass-to-charge (*m/z*) ratios of 32 (^16^O^16^O), 34 (^18^O^16^O), and 40 (Ar) were quantified using membrane inlet mass spectrometry (MIMS) following Ferrón et al. (2016). The system consists of a water inlet, described in detail by Kana et al. (1994), and an analyzer, consisting of a HiQuad^TM^ quadrupole mass spectrometer (QMG 700) with a cross-beam ion source, a Faraday collector, and a 90° off-axis secondary electron multiplier (SEM), connected to a Pfeiffer Vacuum HiCube 80 Eco turbo pumping station. MCR was determined as the difference between GOP and the net O_2_ change during the incubation (Ferrón et al., 2016).

### Glycolate Kinetics and Uptake Experiments

Incubations for glycolate kinetics and diel uptake rates were conducted in semi-transparent acrylic (shaded to approximately match the 15 m depth of sampling) or darkened deckboard incubators flushed with circulating surface seawater to maintain *in situ* temperatures. Incubator bath temperatures were monitored by HOBO Pendant^®^ Data Loggers (Onset Computer Corp.). We refer to samples incubated at simulated light levels of 15 m depth as ‘unshaded,’ and samples incubated in dark conditions as ‘shaded,’ so as not to be confused with nighttime. Accordingly, ‘light’ and ‘dark’ refer to daytime and nighttime.

Experimental procedures for glycolate incubations were described in Casey et al. (2015). Briefly, 60 ml samples were spiked with [U-^14^C]-glycolic acid calcium salt (^14^C-glycolate herein; American Radiolabeled Chemicals, Inc.) at a specific radioactivity of 1.48 TBq mol^-1^. For the kinetics experiment, nine spike concentrations were added, ranging from 1 to 348 nM, spaced logarithmically. For all other incubations, spike concentrations were 10 nM. Uptake time series samples were collected at 4 h intervals for 2 days, Samples were incubated for 4.7 ± 0.4 h. All samples were incubated in triplicate and a 500 μL total activity aliquot was collected from each sample prior to incubation. For glycolate assimilation rates (*v*_A_), samples were filtered under gentle vacuum (<70 mBar) directly after incubation onto 25 mm glass fiber filters (nominal pore size 0.3 μm; GF75, Sterlitech Corp.) and rinsed with three volumes of 20 ml 0.2 μm filtered seawater. Filters were transferred to 20 ml glass scintillation vials and submerged in 10 ml scintillation cocktail (Ultima Gold LLT, Perkin Elmer). To account for ^14^C-glycolate adsorbed to cells or glass fiber filters, a “killed-control” replicate sample poisoned with 2% final concentration paraformaldehyde was included prior to each incubation. Killed-controls were incubated alongside live samples and processed identically. Assimilation depth profiles were conducted alongside PP and MCR *in situ* arrays.

For glycolate respiration rates (*v*_R_), 125 ml glass serum bottles were fitted with rubber sleeve stoppers pierced with center well cups containing a dry piece of fluted cellulose paper (Whatman #2) positioned in the headspace. Respiration incubations were terminated by first soaking the filter paper with 150 μL phenethylamine and then acidifying the sample with 4 ml 4.5 N sulfuric acid through the gas-tight stopper. The acidified sample was allowed to react for at least 48 h before removing the stoppers. This procedure is designed to completely capture the respired ^14^CO_2_. Center well cups were transferred to 20 ml glass scintillation vials and submerged in 10 ml scintillation cocktail. A complete radiochemical mass balance (100 ± 4%) was achieved in the kinetics experiment, and recovery was independent of substrate concentration.

Glycolate uptake rates were calculated as the sum of assimilation and respiration rates. Glycolate-specific energy transduction was calculated as the difference in glycolate respiratory energy yield (*Y*_E_) in shaded and unshaded samples incubated during daylight hours (*Y*_E,Shaded_–*Y*_E,Unshaded_). Glycolate energy yield was calculated as the product of the respiration rate and the standard molar enthalpy of combustion $\left(Y_{E\,\;}\,\;\;=\;v_{R}\times \Delta H_{C}^{\circ }\right)$ of glycolate. Throughout, two-tailed *t*-tests were used to determine significant differences between treatments, and correlation coefficients were determined from model I or model II least-squares regressions, as appropriate for the experimental conditions (Laws, 1997).

## Results

### Glycolate Kinetics Experiment

**FIGURE 1:**
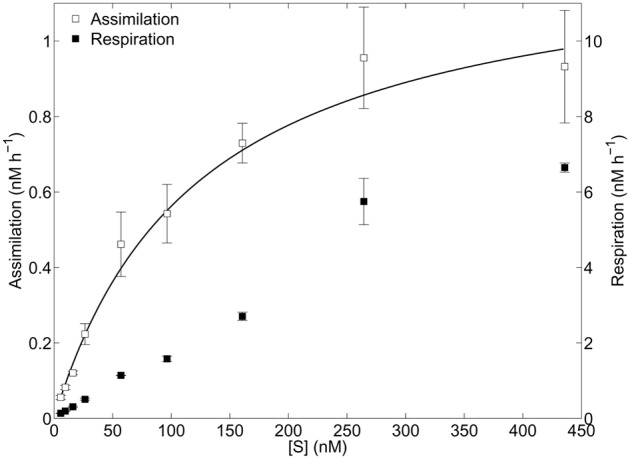
Kinetics experiment. Glycolate assimilation and respiration as a function of added substrate. Error bars represent one standard deviation of the mean rates of three replicates at each concentration. Michaelis–Menten non-linear least-squares regression line is shown (solid line) for assimilation data.

Two distinct kinetics profiles were observed for dark glycolate assimilation and respiration (**Figure 1**). Assimilation followed a monophasic Michaelis–Menten function with a resulting half-saturation concentration (*K*_m,A_) of 118 nM and a maximum velocity (*V*_max,A_) of 1.2 nM h^-1^. Glycolate respiration did not appear to completely saturate over the concentration range tested, therefore *K*_m,R_ and *V*_max,R_ could not be determined. The resulting uptake parameters *K*_m,U_ and *V*_max,U_ were calculated to be 195 and 8.9 nM h^-1^, respectively. Glycolate-specific assimilation efficiency (100^∗^*𝒱*_A_/*𝒱*_A_ + *𝒱*_R_) varied as a logistic function of substrate concentration added (S), with highest efficiencies (29.3 ± 0.9%) corresponding to *S* < 57 nM. At saturating substrate concentrations, the assimilation efficiency approached 12%. The glycolate turnover rate was 0.63 d^-1^, a half-life of 26 h.

### Glycolate Diel Time-Series Experiment

**FIGURE 2:**
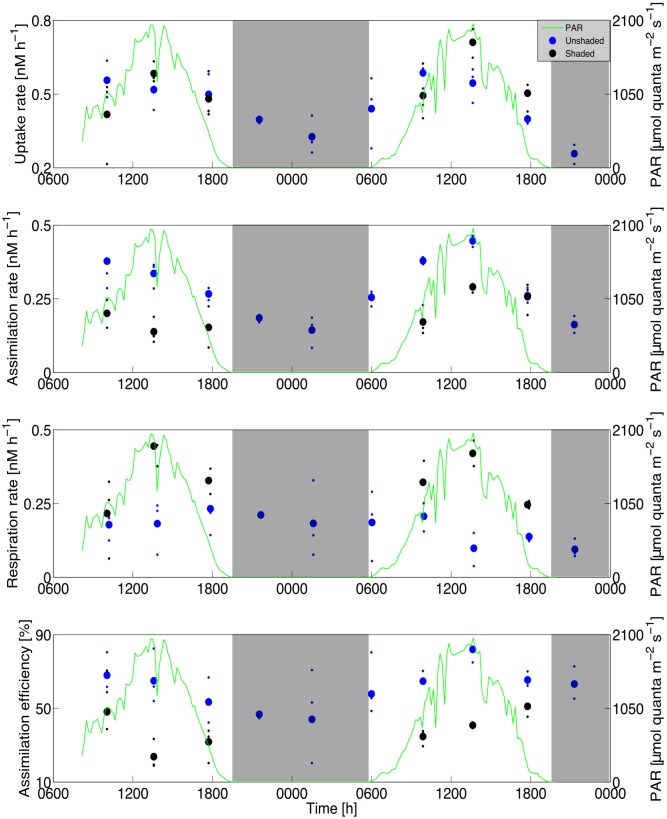
Time-series experiment. Glycolate uptake **(Top)**, assimilation **(Second)**, respiration **(Third)**, and specific assimilation efficiency **(Bottom)** for shaded and unshaded incubations over the course of the diel time-series experiment. Mean (large symbols) and individual data points (small symbols) are shown for clarity. Photosynthetically active radiation (PAR) data are overlaid (green line) in each panel and nighttime is indicated by shaded areas. Data points are aligned to the midpoint of each incubation.

Glycolate uptake rates varied by roughly three-fold (280 ± 70%) over the diel cycle, in phase with the solar cycle, and no difference between shaded and unshaded uptake rates was observed (two-sample *t*-test with unequal variance; *p* = 0.39; **Figure 2**). Glycolate assimilation rates also followed a diel cycle (310 ± 110%), but with maximal rates occurring in unshaded samples in the morning or early afternoon (600–1400 h, local time). Assimilation rates in shaded samples were 35 ± 7% lower than in unshaded samples (*p* = 0.007) during daylight hours, and were similar to nighttime samples (*p* = 0.42). In contrast, glycolate respiration rates in shaded samples were 120 ± 14% higher than unshaded samples (*p* < 0.001) during daylight hours, and unshaded samples were similar over the entire daylength (*p* = 0.71). The discrepancy between daytime light-dependent responses in glycolate assimilation and respiration rates resulted in glycolate-specific growth efficiencies ranging from 24 ± 6% in shaded daytime samples to 82 ± 8% in unshaded daytime samples (*p* < 0.001). Even if the glycolate taken up was quantitatively respired, the maximum potential contribution of phytoplankton uptake of respired ^14^CO_2_ calculated from isotope dilution into the very large dissolved inorganic pool of seawater (approximately 0.006 DPM) would be negligible.

### Glycolate Assimilation Depth Profile Experiment

**FIGURE 3:**
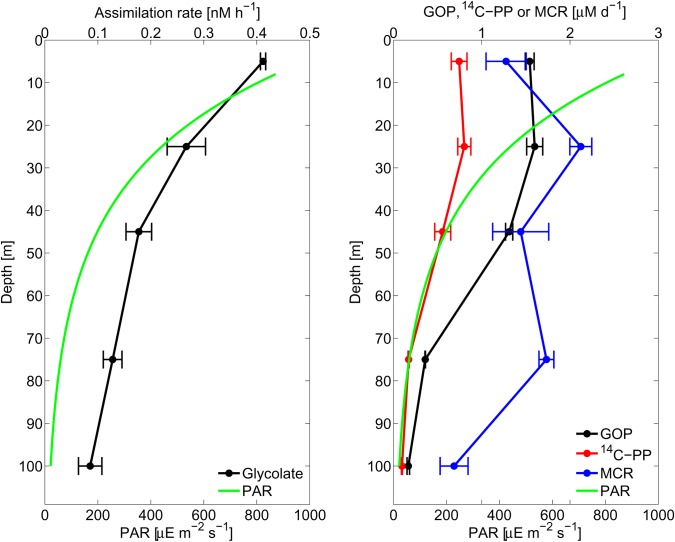
Array profile. Depth profiles of glycolate assimilation rates **(Left)** and productivity [primary production (PP), gross oxygen production (GOP)] and microbial community respiration (MCR) **(Right)**. Carbon units correspond to PP and oxygen units correspond to GOP and MCR. The accompanying PAR profile is shown in both panels. Error bars represent one standard deviation of the mean rates of three replicates at each depth.

Assimilation rates decreased exponentially with depth (**Figure 3**), and were more closely correlated with PAR (Model II geometric mean least-squares fit; *r* = 0.997) than with PP (*r* = 0.88), GOP (*r* = 0.90), or MCR (*r* = 0.51). Importantly, within the mixed layer (36 m; defined as the 0.125 kg m^-3^ offset from 0 m), PP and GOP were uniform, indicating a decoupling of glycolate cycling from PP. In consideration of the differences in incubation lengths between *in situ* and on-deck incubations, assimilation rates at 25 m on the array were similar to average diel time-series assimilation rates (*p* = 0.71).

### Photoheterotrophic Energy Potential and Glycolate Respiration

**FIGURE 4:**
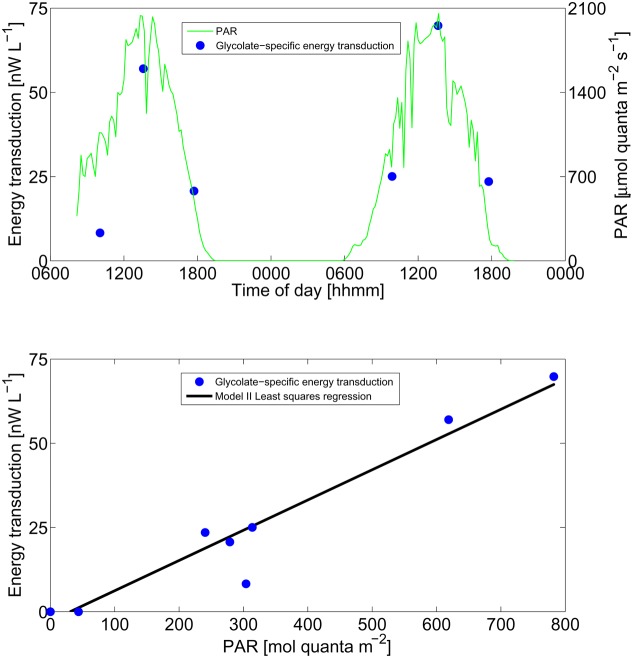
Energy transduction. Calculated glycolate-specific energy transduction (see section “Materials and Methods” for description) and instantaneous PAR irradiance over the diel time-series experiment **(Top)** and as a function of incubation time-integrated PAR **(Bottom)**. Model II geometric mean least-squares regression line is shown.

Energy transduction, such as the pmf generated by PR or BChla-complex, should increase the adenylate energy charge and thus increase the anabolic yield. Similarly, a quantitative substitution of the energy derived from dark glycolate respiration should be derived from light. Since numerous pathways for the assimilation of glycolate are possible, and the composition of each consumer may differ considerably, the free energy of glycolate anabolism cannot be estimated reliably. Instead, we introduce a quantity derived from the enthalpy generated by the respiration of glycolate in shaded and unshaded incubations during the daytime. This quantity, glycolate-specific energy transduction, reflects the maximum chemical potential energy derived from light-dependent processes. Glycolate-specific energy transduction was linearly correlated with PAR integrated over each daytime incubation (*r* = 0.97; *p* < 0.001; **Figure 4**). Accordingly, over the range of irradiances observed, the relationship between these two energy quantities (light and chemical energy) did not saturate.

## Discussion

Glycolate, a low molecular weight (76 Da) hydroxy acid, may constitute an important flux of both carbon and energy within the marine microbial community metabolism. Dark glycolate uptake kinetics indicated an upper bound ambient concentration of 30 ± 6 nM, based on turnover time as a function of substrate added (Dietz et al., 1977). This estimate, though an upper bound (Laws, 1983), is roughly half the nighttime concentration measured by HPLC at an oligotrophic site (60–70 ng Chl a L^-1^) in the tropical eastern North Atlantic (66 nM; Leboulanger et al., 1997), and is well below the *K*_m,U_ (195 nM). During the dark kinetics incubation, the glycolate pool turned over approximately daily (1.1 ± 0.1 days); however, this is likely an underestimate due to disequilibrium with respect to production (which is exclusively during daylight hours).

Time-series incubations showed a characteristic diel cycle in uptake rates in phase with the solar cycle, independent of whether the sample was exposed to light. Taken together with our understanding of a photorespiratory source of glycolate, these results indicate ambient concentration-dependent rates, rather than light-enhanced uptake by heterotrophs. Although the time-series experiment and kinetics experiment were conducted on separate expeditions with somewhat different phytoplankton community stocks and rates (Cruise 1: Chl *a* = 80 ng L^-1^, PP = 8.1 mg C L^-1^ d^-1^; Cruise 2: Chl *a* = 137 ng L^-1^, PP = 9.9 mg C L^-1^ d^-1^), nighttime uptake rates (10 nM spike) collected during the time-series experiment closely matched the corresponding values from the kinetics curve (*p* = 0.81). With this caution, we conservatively estimate a two-fold change in ambient glycolate concentrations over the course of the diel cycle, which is consistent with estimates from the North Atlantic (2.4 ± 1.2-fold; Leboulanger et al., 1998). Glycolate-specific respiration rates accounted for approximately 3% of total community oxygen consumption (respiration rates), and considering the glycolate respiration quotient, 6% of total community respired CO_2_ (assuming a total community respiration quotient of 1.0; del Giorgio et al., 2006; c.f., Berggren et al., 2011). On a carbon basis, glycolate production rates accounted for less than 4% of GOP in the time-series incubations, however, this can also be interpreted as an underestimate of gross photorespiration since salvage pathways are present in some photoautotrophs. Due to methodological challenges, photorespiration rates have only been indirectly measured in the oceans (Carrillo et al., 2004), and may be an important but largely ignored flux of carbon (Karl et al., 1996).

High-light adapted ‘ecotypes’ (eHL) of the marine cyanobacterium *Prochlorococcus*, the most abundant photoautotroph at Station ALOHA, lacks glycolate oxidase or glycolate dehydrogenase, an essential step in the salvage pathway for photorespiratory glycolate regeneration of 3-phosphoglycerate. Because the precursor 2-phosphoglycolate is toxic to central carbon fixation pathways, *Prochlorococcus* actively excretes glycolate via an ATP-binding cassette efflux transporter. In cultures of two eHL *Prochlorococcus* strains, glycolate excretion was 3% of carbon fixation (Bertilsson et al., 2005), remarkably close to our upper bound estimate. It should be noted that the diazotrophic cyanobacterium *Crocosphaera*, which was relatively abundant during the time-series expedition (100–700 cells ml^-1^; 3% of PP; calculated from Wilson et al., 2017), does have a complete photorespiratory salvage pathway. Therefore we cannot eliminate the possibility that *Crocosphaera* could take up glycolate. Notwithstanding, *Prochlorococcus* was the dominant primary producer during the time-series expedition, and was likely the major glycolate producer. Interestingly, a major consumer of glycolate is likely the numerically dominant heterotroph at Station ALOHA, SAR11, a small alphaproteobacterium with an absolute growth requirement for pyruvate and either glycolate or one of several photorespiratory salvage pathway intermediate metabolites. SAR11 has both a glycolate transporter and glycolate oxidase which yields glyoxylate and hydrogen peroxide. In addition to the apparent co-evolution of these two dominant oligotrophs (Braakman et al., 2017), SAR11 and much of the heterotrophic microbial community at Station ALOHA (Rusch et al., 2007), have genes for proteorhodopsin-based phototrophy, prompting our investigation into the light-dependent metabolism of glycolate. With a respiration quotient (mol CO_2_: mol O_2_) of 2, a carbon redox number of 3, and a standard carbon molar enthalpy of combustion (ΔH°_c_) of 340 KJ [C-mol]^-1^, glycolate is a relatively poor energy substrate. Accordingly, heterotrophic growth on carboxylic and hydroxy acids like acetate and fatty acids typically requires the operation of the glyoxylate shunt (Kornberg, 1966), a bypass of two CO_2_ evolving steps of the oxidative tricarboxylic acid pathway by way of isocitrate lyase and malate synthase which allows the net accumulation of carbon through acetyl-CoA. However, at least one alternative pathway utilizing glyoxylate is present in SAR11 and many other heterotrophs, consisting of a heterotrophic analog of the photorespiratory salvage pathway which can supply precursors for gluconeogenesis (by way of 3-phosphoglycerate) or a number of amino acid synthesis pathways (by way of L-glycine). These anabolic pathways cannot be sustained without a supplemental energy source, since the ATP/NAD(P)H ratio and yield of the glyoxylate shunt using glycolate as a substrate does not satisfy the demands of e.g., protein synthesis (calculation based on *i*AF1260, a metabolic model of *Escherichia coli* K-12 MG1655; Feist et al., 2007).

The central finding of this study, that exposure to light enhances the glycolate-specific assimilation efficiency, points to the possibility that the pmf generated by PR or by the BChla-complex yields sufficient energy to divert glycolate flux from the mostly catabolic glyoxylate shunt to the mostly anabolic pathways. We cannot eliminate the possibility that another light-dependent process which is decoupled from PP and community respiration could reproduce our observations. However, the strong correspondence between light and glycolate-specific assimilation efficiency, independent of concentration, supports the notion that photoheterotrophy supplements cellular energy demands for growth on oxidized substrates. Furthermore, considering the ΔH°_c_ and the maximum chemical potential energy yield of glycolate respiration, the resulting energy yield was closely correlated with PAR irradiance integrated over each incubation, rather than PP or community respiration. Unfortunately, it is not possible to ‘scale’ glycolate-specific phototrophic energy yields to total photoheterotrophy, since the composition of the myriad additional growth substrates and their respective uptake rates and light-dependent growth efficiencies is not known. We suggest that light-enhanced biomass yields may play an important role in the co-evolution of *Prochlorococcus* and SAR11 (Braakman et al., 2017), a metabolic coupling supported by the exchange of low-yield, thermodynamically optimal substrates (glycolate and pyruvate; thermodynamic efficiency = 20–24%; Westerhoff et al., 1983).

## Author Contributions

JRC, SF, and DMK planned the experiments, JRC and SF collected and processed the samples and analyzed the data. All authors contributed to the manuscript preparation and editing.

## Conflict of Interest Statement

The authors declare that the research was conducted in the absence of any commercial or financial relationships that could be construed as a potential conflict of interest.

## Footnotes

1 http://hahana.soest.hawaii.edu/hot/methods/results.html
